# To: Critical COVID-19 and neurological dysfunction - a direct comparative analysis between SARS-CoV-2 and other infectious pathogens

**DOI:** 10.62675/2965-2774.20240291-en

**Published:** 2024-05-27

**Authors:** Roberto A. Villa

**Affiliations:** 1 Hospital General de Agudos Juan A. Férnandez Intensive Care Unit Buenos Aires Argentina Intensive Care Unit, Hospital General de Agudos Juan A. Férnandez - Buenos Aires, Argentina.

**To the Editor**

We have read with great care the interesting article by Teixeira-Vaz et al. on a prospective, single-center cohort study of 27 coronavirus disease 2019 (COVID-19) patients requiring mechanical ventilation for >48 hours for acute respiratory distress syndrome (ARDS).^(1)^ They noted that although neurological impairment caused by severe acute respiratory syndrome coronavirus 2 (SARS-CoV-2) infection usually occurs in a variable manner, almost all patients with respiratory failure and ARDS develop impairment. Although information is limited, as Finsterer and Scorza noted,^(2)^ we support the need for a prospective study on patients with neurological dysfunction after severe SARS-CoV-2 infection. In addition, we agree with the points raised by Magoon and Suresh.^(3)^

Patients with COVID-19 and severe respiratory compromise suffer hypoxemia-induced brain injury, which manifests as cognitive deficits in acute illness and during long-term follow-up. This result is not unusual since SARS-CoV-2 is a neurotropic virus that causes neuroinvasion via several potential mechanisms, including transsynaptic transfer, olfactory nerve entry, vascular endothelial infection, and leukocyte migration across the blood-brain barrier.^(4)^ Additionally, using a Trojan horse mechanism, the virus can infect T lymphocytes, which can then cross the blood-brain barrier and infect the CNS.^(5)^ The final consequence of viral invasion of the nervous system in severe SARS-CoV-2 infections is septic encephalopathy, which is a type of brain failure caused by inflammation with endothelial/microglial activation, increased permeability of the blood-brain barrier, hypoxia, an imbalance of neurotransmitters, glial activation, and axonal and neuronal loss.^(6)^ COVID-19-associated encephalopathy is multifactorial and involves hypoxic changes, intracranial pressure changes, and viral responses in brain tissue.^(7)^ This process also occurs in patients with persistent or prolonged COVID-19. The current theory of the mechanism underlying prolonged COVID-19 is that the inflammatory process promoted by SARS-CoV-2 infection disrupts the blood-brain barrier in patients with neurological involvement. The fact that proinflammatory cytokine levels do not predict long-term functional outcome suggests that prognosis is more closely related to neuronal damage than to the acute neuroinflammatory process.^(8)^ In each of these situations, a potential neuroprotective factor may play a crucial role in preserving neuronal tissue.^(9)^

Among our series of 468 patients with COVID-19 and critical respiratory failure with ARDS, 99% of recovered patients had cognitive deficits as measured by the sum of the Montreal Cognitive Assessment, Barthel Index, Beck Depression Inventory II, Beck Anxiety Inventory, Richard Campbell Sleep Questionnaire and Pittsburgh Sleep Quality Index scores. Interestingly, we treated previously healthy individuals with acute-onset viral disease, severe respiratory failure, and variable neurological compromise at the time of recovery, from a mild cognitive deficit to severe impairment with a minimally conscious state.

We believe that closely examining the neurological damage caused by SARS-CoV-2 in critically ill patients is of fundamental importance. However, this interesting study has limitations that call the results into question and complicate their interpretation. Comprehensive clinical investigations are necessary to make an accurate diagnosis and initiate early and appropriate treatment in critical COVID-19 patients since the vast majority have neurological complications.
