# Supplementary material for: To: Critical COVID-19 and neurological dysfunction - a direct comparative analysis between SARS-CoV-2 and other infectious pathogens
Source: Crit Care Sci. 2024 May 27;36:e20240291en. doi: 10.62675/2965-2774.20240291-en (PMC11152443; doi:10.62675/2965-2774.20240291-en)
Supplement: Translation

# Translation: Para: COVID-19 crítico e disfunção neurológica – uma análise comparativa direta entre o SARS-CoV-2 e outros patógenos infecciosos

**DOI:** 10.62675/2965-2774.20240291-pt

**Authors:** Roberto A. Villa

**Affiliations:** 1 Buenos Aires Argentina Unidade de Terapia Intensiva Hospital General de Agudos Juan A. Férnandez - Buenos Aires, Argentina.

**Ao Editor**

Lemos com muita atenção o artigo de Teixeira-Vaz et al., um estudo de coorte prospectivo unicêntrico envolvendo 27 pacientes com a doença do coronavírus 2019 (COVID-19) que necessitaram de ventilação mecânica por mais de 48 horas devido à síndrome do desconforto respiratório agudo (SDRA).^(1)^ Os autores observaram que, embora o comprometimento neurológico causado pela infecção pelo coronavírus da síndrome respiratória aguda grave 2 (SARS-CoV-2) geralmente se apresente de forma variável, quase todos os pacientes com insuficiência respiratória e SDRA desenvolvem algum grau de comprometimento. Embora as informações sejam limitadas, como ressaltam Finsterer e Scorza,^(2)^ acreditamos que há necessidade de um estudo prospectivo de pacientes que desenvolveram disfunção neurológica após infecção grave pelo SARS-CoV-2. Além disso, concordamos com os pontos levantados por Magoon e Suresh.^(3)^

Pacientes com COVID-19 e comprometimento respiratório grave sofrem hipóxia cerebral, que se manifesta como défice cognitivo durante a doença aguda e no acompanhamento a longo prazo. Esse resultado não é incomum, uma vez que o SARS-CoV-2 é um vírus neurotrópico que causa neuroinvasão por meio de diversos potenciais mecanismos, incluindo transferência transsináptica, entrada pelo nervo olfatório, infecção endotelial vascular e migração de leucócitos através da barreira hematoencefálica.^(4)^ Além disso, utilizando um mecanismo de cavalo de Troia, o vírus pode infectar linfócitos T, que, então, atravessam a barreira hematoencefálica e infectam o sistema nervoso central.^(5)^ A consequência final da invasão viral do sistema nervoso em infecções graves por SARS-CoV-2 é a encefalopatia séptica, que é um tipo de disfunção cerebral causada por inflamação com ativação endotelial/microglial, aumento da permeabilidade da barreira hematoencefálica, hipóxia, desequilíbrio de neurotransmissores, ativação glial e perda axonal e neuronal.^(6)^ A encefalopatia associada à COVID-19 é multifatorial e envolve alterações hipóxicas, alterações da pressão intracraniana e respostas virais no tecido cerebral.^(7)^ Esse processo também ocorre em pacientes com COVID-19 persistente ou prolongada. A atual teoria do mecanismo subjacente à COVID-19 prolongada é a de que o processo inflamatório promovido pela infecção por SARS-CoV-2 afeta a barreira hematoencefálica em pacientes com comprometimento neurológico. O fato de que os níveis de citocinas pró-inflamatórias não predizem desfechos funcionais a longo prazo sugere que o prognóstico está mais relacionado ao dano neuronal do que ao processo neuroinflamatório agudo.^(8)^ Em cada uma dessas situações, um potencial fator neuroprotetor pode desempenhar papel crucial na preservação do tecido neuronal.^(9)^

Na nossa série de 468 pacientes com COVID-19 e insuficiência respiratória crítica com SDRA, 99% dos que se recuperaram apresentaram défices cognitivos, conforme medido pela soma dos escores da *Montreal Cognitive Assessment*, do índice de Barthel, do Inventário de Depressão de Beck – Segunda Edição, do Inventário de Ansiedade de Beck, do *Richards-Campbell Sleep Questionnaire* e do Índice de Qualidade do Sono de Pittsburgh. É importante ressaltar que tratamos indivíduos previamente saudáveis com doença viral de início agudo, insuficiência respiratória grave e comprometimento neurológico variável no momento da recuperação (desde défice cognitivo leve até comprometimento grave com estado minimamente consciente).

Acreditamos que examinar de perto o dano neurológico causado pelo SARS-CoV-2 em pacientes críticos é extremamente importante. No entanto, este estudo interessante possui limitações que questionam os resultados e complicam sua interpretação. Investigações clínicas abrangentes são necessárias para fazer um diagnóstico preciso e iniciar o tratamento precoce e apropriado de pacientes críticos com COVID-19, uma vez que a maioria apresenta complicações neurológicas.
